# Emerging Strategies in TCR-Engineered T Cells

**DOI:** 10.3389/fimmu.2022.850358

**Published:** 2022-03-30

**Authors:** Fang Wei, Xiao-Xia Cheng, John Zhao Xue, Shao-An Xue

**Affiliations:** Genetic Engineering Laboratory, School of Biological & Environmental Engineering, Xi’An University, Xi’An, China

**Keywords:** T cell receptor, genetic engineering, cancer immunotherapy, TCR-engineering, new strategies

## Abstract

Immunotherapy of cancer has made tremendous progress in recent years, as demonstrated by the remarkable clinical responses obtained from adoptive cell transfer (ACT) of patient-derived tumor infiltrating lymphocytes, chimeric antigen receptor (CAR)-modified T cells (CAR-T) and T cell receptor (TCR)-engineered T cells (TCR-T). TCR-T uses specific TCRS optimized for tumor engagement and can recognize epitopes derived from both cell-surface and intracellular targets, including tumor-associated antigens, cancer germline antigens, viral oncoproteins, and tumor-specific neoantigens (neoAgs) that are largely sequestered in the cytoplasm and nucleus of tumor cells. Moreover, as TCRS are naturally developed for sensitive antigen detection, they are able to recognize epitopes at far lower concentrations than required for CAR-T activation. Therefore, TCR-T holds great promise for the treatment of human cancers. In this focused review, we summarize basic, translational, and clinical insights into the challenges and opportunities of TCR-T. We review emerging strategies used in current ACT, point out limitations, and propose possible solutions. We highlight the importance of targeting tumor-specific neoAgs and outline a strategy of combining neoAg vaccines, checkpoint blockade therapy, and adoptive transfer of neoAg-specific TCR-T to produce a truly tumor-specific therapy, which is able to penetrate into solid tumors and resist the immunosuppressive tumor microenvironment. We believe such a combination approach should lead to a significant improvement in cancer immunotherapies, especially for solid tumors, and may provide a general strategy for the eradication of multiple cancers.

## Introduction

Immunotherapy of cancer based on adoptive cell transfer (ACT) of T lymphocytes can be classified into three approaches. The first, tumor-infiltrating lymphocyte (TIL) therapy, harvests naturally occurring T cells that have already penetrated patient tumors, expands them *ex vivo*, and then re-infuses them into patients (1, 2). However, it is often very difficult to isolate tumor-specific TILs, which are not present in all patients or may generate too few cells for therapeutic efficacy.

The second approach, chimeric antigen receptor (CAR)-modified T cells (CAR-T), bypasses this problem by directly engineering T cells with known tumor-specific CARs. CARs are fusion molecules that link a single-chain antibody with T-cell activation signaling domains such as CD28-CD3ζ (3) or 4-1BB-CD3ζ (4). When a CAR is transduced into human T cells, the antibody fragment is expressed on the surface of the engineered T cells to recognize a tumor antigen expressed on tumor cells, while the CD28-CD3ζ or 4-1BB-CD3ζ domain delivers a stimulatory signal once the antibody binds to a tumor antigen, activating CAR-T cells to attack the tumor.

CAR-T cells are not restricted by MHC molecules, and thus one CAR-T construct can be used to treat any patient regardless of genetic background. Currently, the most widely used and successful CAR is the CD19-CAR, which recognizes the CD19 molecule expressed on the surface of B cells, thus can eliminate some B-cell-derived leukemias and lymphomas (5, 6), including complete response in nearly 90% of B-cell leukemia patients (5). However, antibody-based CARs can only recognize antigens expressed on the cell surface, and not intracellular antigens, limiting the number of targets and potential tumor types addressable by CAR-T therapy.

The third approach, T cell receptor (TCR)-engineered T cells (TCR-T), uses TCRS as found on native T cells to confer specificity, instead of antibody-based CARs. TCRS can be isolated from tumor-reactive T cells and further modified for enhanced expression and functions. TCRS can recognize both cell-surface and intracellular targets, these include neoantigens (neoAgs) that arise from mutations and are specific to tumor cells. The disadvantage of using TCR-T is that TCRS are restricted by MHC molecules, thus any given TCR can only be used to treat patients with the corresponding MHC genetic background. In the following sections, we describe the settings in which TCR-T may prove most effective.

## TCR-T Immunotherapy for Cancer as a Complement to CAR-T

CAR-T has been most successful in hematological malignancies (7–10), with FDA-approved therapies targeting CD19 (Kymriah, Yescarta, Tecartus, Breyanzi) and BCMA (Abecma) as of December 2021. CD19-CAR-T can achieve complete response in nearly 90% of B cell leukemia patients, although 50% of patients may nonetheless relapse (11). One of the major reasons for this relapse is loss of surface expression of CD19 from tumor cells (12), thus evading recognition by CD19-CAR-T cells. These patients may no longer respond to CD19-CAR-T, although targeting different antigens may still be viable, in which case TCR-T may be used as a late-line option. For example, a recent study reported transfer of WT1-TCR-engineered donor T cells into AML patients at high risk of relapse following allogeneic stem cell transplantation, 12/12 treated patients achieved relapse-free survival (13), compared to 54% in a concurrent group of 88 similar high-risk patients, and WT1-TCR-T cells also showed prolonged persistence and maintenance of antigen-specific polyfunctional activity.

The greater opportunity for TCR-T may exist in solid tumors, where CAR-T has been less effective (14–16). The mechanisms behind these limitations are poorly understood and under active investigation. CAR-T recognition is limited to surface antigens, and moreover CAR-T activation requires higher concentration of target antigens (17, 18). This lower sensitivity helps avoid damage to normal tissues with low antigen expression (19), but conversely may be unsuitable for tumors with similarly low tumor antigen expression. For example, CAR-T specific for anaplastic lymphoma kinase (ALK) showed variable efficacy towards different cell lines depending on expression level of ALK (17). Recently, a study investigated B cell malignancies with up to 33-fold lower CD20 expression than healthy B cells – below the concentration required to activate CAR-T – but found that CD20-specific TCR-T clones with high avidity were able to overcome self-tolerance and eliminate these tumor cells (20). It is estimated that CAR-T cells need in the order of hundreds of target molecules to be activated (17, 18, 21), whereas TCR-T can be activated by a single target molecule (22).

Initial clinical studies of TCR-T in solid tumors have shown promising results (23, 24). An affinity-enhanced NYESO1-TCR achieved 45-55% clinical response rate in metastatic melanoma patients (25, 26), and 50-61% clinical response rate in metastatic synovial sarcoma patients (25–27). The same NYESO1-TCR achieved 80% clinical response rate in multiple myeloma patients without apparent side effects, including 70% complete response rate with median progression-free survival of 19 months (28). Recently, a phase 1 trial of TCR-T targeting HPV-16 E7 in metastatic HPV-associated epithelial cancers achieved 50% clinical response rate (6/12 patients), including 4/8 patients refractory to PD-1 blockade (29).

In recent years, neoAgs have been discovered as a class of immunogenic tumor-specific antigens that are derived from tumor-specific mutations of self-proteins (30–32) or from tumor-causing oncogenic viral proteins (33) in the estimated 15% of human cancers attributed to viruses (34). T cells specific for neoAgs and viral proteins would not have undergone central thymic tolerance selection, making it possible to isolate high-avidity T cell clones against these targets. These antigens are rarely expressed on the cell surface, and represent a therapeutic opportunity in solid tumors using TCR-T that CAR-T may be unable to match (35).

Despite these promising results (24–27, 29), several hurdles remain to be overcome to realize the true promise of TCR-T immunotherapy. In early clinical trials, some non-responder patients lacked *in vivo* persistence of the infused T cells (36, 37), suggesting that the transferred TCR-T cells need additional support to enhance their *in vivo* survival. Some patients with late relapse showed no evidence of T cell infiltration in the tumor, and moreover infused TCR-T cells face a hostile immunosuppressive tumor microenvironment (TME). In the following sections, we discuss current and future engineering strategies to address these challenges to deliver effective and long-lasting tumor control to a broad range of cancer patients.

## Overcoming the Challenges of TCR-T Cancer Immunotherapy

### Enhancing TCR Expression and Function Through Reducing TCR Mis-Pairing

TCR-T therapy relies on mRNA or viral transduction of tumor-reactive TCR genes to redirect T cell specificity towards tumor cells. However, using either mRNAs or randomly integrating viruses to deliver the exogenous TCR, leaves the endogenous TCR genes intact. Therefore, this could potentially result in some degree of mis-pairing between the introduced and endogenous TCR chains (38–40). Mis-pairing poses certain safety risks, as T cells expressing mis-paired TCRS may be auto-reactive against the patient’s MHC molecules. Indeed, a murine study showed that TCR mis-pairing in the context of adoptive transfer of TCR-gene-modified T cells combined with increased conditioning resulted in graft-versus-host disease (GvHD) and serious animal death (41). Similarly, an *in vitro* study of human EBV-transformed lymphoblastoid cell lines showed that mis-paired TCRS drove potentially dangerous off-target toxicity (42).

Several strategies have been explored to prevent TCR mis-pairing (43, 44). The interaction between TCRα and TCRβ chains is largely governed by the invariant Cα/Cβ-interface (45), enabling modification of this region to prevent pairing with endogenous TCRS. Reciprocal “knob-hole” amino acid changes in the center of the TCR C domains led to preferential pairing of the modified chains while disfavoring combinations with native TCR chains (46). Introduction of an additional inter-chain disulfide bond within the TCR Cα/Cβ-interface (47) also enhanced the pairing of the modified chains whilst reducing the efficiency of pairing with wild-type chains (48, 49). This preferential pairing of cysteine-modified TCR chains has accounted for improved TCR gene expression and enhanced antitumor activity of transduced T cells (50). Replacing the human TCR constant domains with whole (49, 51, 52) or partial (53, 54) murine sequences represents an alternative strategy to reduce unwanted mis-pairing, and can also increase the expression level of the introduced TCR genes (51). The enhanced expression of the human/murine hybrid TCR in human T cells may be partly due to the greater binding capacity of the murine TCR constant domains to human CD3 molecules when compared with human TCR constant domains (51). Finally, instead of using murine sequences, exchanging the human TCR constant domains Cα and Cβ with each other (domain swapping), or replacing Cα and Cβ with the corresponding γδ TCR constant domains, could also generate functional TCRS with reduced mis-pairing and improved safety profile (55). However, it is important to note that the individual TCR subfamily V-domains and even the antigen-binding CDR3 α/β-loops may also contribute to the interaction of TCR α and β chains (56, 57), and hence manipulation of the TCR constant domains can only partially reduce the frequency of mis-pairing, rather than eliminate the risk completely.

Another common approach to reduce mis-pairing is to generate a so-called single-chain TCR (Sc-TCR) by covalently linking the Vα and Vβ domains with a polylinker (PLK), resulting in a single polypeptide which will in theory inhibit mis-pairing through steric hindrance (58). T-cell-activation signaling upon antigen encounter is provided by fusion of CD3ζ onto the Cβ-chain within the Sc-TCR. Using this approach, Sebestyen et al. showed preferential pairing between CD3ζ-modified TCR α and β chains while reducing mis-pairing with unmodified TCR chains (59). To develop this concept further, Aggen et al. replaced the constant domains of the Sc-TCR construct with a CD28 or 4-1BB together with CD3ζ or LCK signaling domains (60). Although this strategy was able to reduce mis-pairing, activation of these T cells upon antigen encounter no longer followed natural TCR signaling pathways, but rather that of conventional CARs. Because CAR signaling is less efficient than that of the TCR complex (23, 61), Voss et al. developed an alternative Sc-TCR scaffold Vα-PLK-Vβ-Cβ plus Cα (62), relying on assembly with the native CD3 complex for more physiologic T-cell signaling. To stabilize the structure of the Sc-TCR, we introduced an extra new disulfide bond between the Vα and the polylinker, which strengthens the interaction between the Vα and Vβ domains, favoring surface expression of the Sc-TCR, while also greatly reduced TCR mis-pairing (63). One of the potential drawbacks of using this technology is that not all of the TCRS can form a stable Sc-TCR. According to our experience, some of the weak TCRS can not be expressed on the surface of T cells as a Sc-TCR due to the weak interaction between the Vα and Vβ domains. With such weak TCR, genetic engineering of certain frame work regions of the TCR may be able to help to resolve the problem (64).

Aside from TCR protein design, another way of reducing TCR mis-pairing and its related side effects is to knock out the endogenous TCRS *via* genetic engineering, which also reduces competition for CD3 binding from endogenous TCRS (65). Several strategies have been explored to achieve this goal, including the use of siRNAs (66, 67), zinc-finger nucleases (68), transcription activator-like effector nucleases (TALENs) (69, 70), and CRISPR/Cas9 technologies (71, 72). As the CRISPR/Cas9 has several advantages, including (i) simple and highly efficient editing, (ii) rapid and affordable manufacturing, (iii) versatile multiplex genome editing through simultaneously targeting several genes, and (iv) user-friendly and easily deliverable. Therefore, this CRISPR/Cas9 system holds great promise and may lead the way for future genetic engineering of T cells for cancer immunotherapy (73). The feasibility of genome editing using CRISPR/Cas9 targeting the TRAC and TRBC loci was recently demonstrated in primary T cells (71, 74). Using multiplex genome editing, the beta-2 microglobulin class I MHC and PD-1 genes can also be disrupted alongside the TCR α and β genes (75, 76). Removal of endogenous TCR and class I MHC eliminates allogeneic antigen recognition and reduces risk of GvHD and donor T cell rejection, generating allogeneic ‘universal’ T cells that can be infused in any recipient (77, 78), as opposed to autologous T cells that can only be re-infused into the donor patient. The immune checkpoint gene PD-1 is removed to enhance T cell activity, for which reason the T cell suppressor LAG-3 has also been knocked out to improve antitumor activity *in vitro* and in murine xenografts (79). We anticipate that other immune inhibitory receptors such as TGFβ receptors can also be disrupted to generate universal CAR-T and TCR-T cells with enhanced resistance to the inhibitory TME.

TCR-T cells have been generated by TCR transduction of T cells in which the endogenous TCR α chain (80), β chain (81), or both α and β chains (82) were removed using CRISPR/Cas9, or by orthotopic replacement of TCR-αβ chains with tumor reactive TCRS using CRISPR/Cas9 (83, 84), resulting in engineered TCR-T cells with enhanced TCR expression and prolonged control of tumor growth in preclinical murine models. However, for clinical use, the potential off-target toxicity of the CRISPR/Cas9 technology has to be taken into considerations (85). A recent study showed that DNA breaks introduced by sgRNA/Cas9 can lead to on-target mutagenesis, such as large deletions and genomic rearrangements at the targeted sites in mouse embryonic stem cells and in a human differentiated cell line (86). Therefore, strategies for improving safety of the CRISPR/Cas9 technology need to be put in place. To reduce the off-target toxicity, high-fidelity CRISPR–Cas9 nuclease has been developed (87), and a recent review has provided many strategies about how to refine the CRISPR/Cas9 technology for clinical applications (88). Recently, a phase 1 clinical trial was designed to test multiplex CRISPR-Cas9 gene editing of T cells from patients with advanced, refractory cancer (89), in which endogenous TCR α and β chains were removed to prevent mis-pairing, and PD-1 removed to avoid T cell exhaustion. The NYESO1-TCR engineered T cells persisted for up to 9 months and trafficked to tumor sites, demonstrating proof-of-concept for multiplex CRISPR gene editing in cell therapy. Another study applied CRISPR-edited T cells in patients with refractory non-small-cell lung cancer also concluded that clinical application of CRISPR–Cas9 gene-edited T cells is generally safe and feasible (90).

To achieve the best results of maximally reducing mis-pairing and enhancing expression and function of the tumor-specific TCR, we have recently combined multiple strategies by knocking out endogenous TCR using CRISPR/Cas9 together with transduction of a single-chain EBV-specific TCR (EBV-Sc-TCR) (91). This almost eliminated mis-pairing between the introduced EBV-Sc-TCR and endogenous TCR chains, and we further enhanced tumor-specific TCR expression, functional avidity, and IL-2 production by introducing an extra intra-chain disulfide bond between the Vα and the poly-linker (91).

### Enhancing Persistence and Anti-Tumor Functions of the Genetically Engineered T Cells

T cell persistence is a fundamental requisite for durable immunosurveillance, as many clinical trials revealed that most non-responder patients showed no *in vivo* persistence of the infused tumor specific T cells (36, 37), and in contrast, patients who achieved complete response or relapse-free survival and tumor control showed robust proliferative capacity and long-term persistence of engineered T cells (13, 27). To maintain persistence of the transferred T cells, a variety of cytokines have been coadministered to support T cell survival and expansion. The standard ACT regimen comprises lymphodepletion with cytotoxic agents, including cyclophosphamide and fludarabine, followed by administration of recombinant human IL-2 after T cell transfer (92). Systemic delivery of IL-2, is known to expand T cells while maintaining functional activity (93), has achieved durable regression in some metastatic melanoma and renal cancer patients (94), is approved by the FDA, and is used in both CAR-T and TCR-T immunotherapy of cancers today. However, there is evidence suggests that IL-2 may preferentially expand CD4^+^ regulatory T (Treg) cells rather than tumor-killing CD8^+^ cytotoxic T cells (CTLs) (95, 96). Therefore, recent attention has focused on modifying the IL-2 molecule to preferentially bind and activate CD8**^+^** CTLs over Treg cells (97). For example, a half-life-extended super mutant IL-2 conjugated to a tumor-targeting antibody allowed more efficient CTL stimulation and expansion in the TME, resulting in significantly improved complete response rate and lower tumor relapse *in vivo* (97).

IL-7, is a hematopoietic cytokine regulating multiple aspects of T cell biology (98), is essential for T cell survival and homeostatic proliferation, and promotes the survival of naïve and memory T cells by upregulating the antiapoptotic molecule Bcl-2 (99, 100). IL-7 supplementation improved the persistence and efficacy of transferred T cells, supporting its usage as an adjuvant for adoptive immunotherapy (101). When IL-7 was co-expressed in NKG2D-based CAR-T cells, it enhanced CAR-T persistence and expansion while inhibiting apoptosis and exhaustion (102). Similarly, IL-7 co-expression in GPC3-CAR-T cells improved CAR-T efficacy toward liver cancer (103).

IL-12 is a major contributor to effective anti-tumor immune responses (104), stimulating the effector functions of activated T cells and NK cells *via* induction of cytotoxic enzymes such as perforin and cytokines such as IFN-γ (104, 105). Cytotoxic enzymes can mediate direct killing of tumor cells (106), while production of IFNγ from NK cells as well as CD4^+^ and CD8^+^ T cells inhibits tumor growth (107, 108). IL-12 further modifies the TME through inhibition of Tregs (107), upregulating MHC class I presentation on tumor cells (109, 110), and converting immunosuppressive M2 macrophages into activated antitumor M1 macrophages (111). IL-12 also prevents the activation-induced cell death of naïve CD8^+^ T cells, favoring their survival and differentiation towards the effector phenotype to sustain anti-tumor activity against mouse models of melanoma (112).

These studies demonstrate that IL-12 is not only required for the activation of anti-tumor cytotoxic immune responses, but also directly relieves immune suppression (107). However, systemic administration of IL-12 is very toxic (113), severely limiting its utility in clinical applications. To minimize systemic exposure and potential toxicity while maintaining the beneficial effects of IL-12, several strategies have been explored, for example local delivery (114, 115), encapsulating IL-12 with nanoparticles or heparin (116, 117). Alternatively, deleting the N-terminal signal peptide of IL-12 or tethering IL-12 to the surface of TCR-engineered T cells *via* a membrane anchor prevents secretion (118, 119), thereby attenuating toxicity while improving antitumor efficacy. These treatment strategies may have broad applications to cellular therapy with TILs, CAR-T, and TCR-T cells. A recent multi-center phase 1 trial used a chemically activatable IL-12 gene delivered into the tumor site, where IL-12 expression triggered by the drug veledimex achieved conversion of an immunologically “cold” TME to an inflamed “hot” TME with increased influx of IFN-γ–producing T cells (120). IL-18 is another cytokine that shares biological effects with IL-12 but with reduced toxicity (121). Recent studies exploring IL-18 in the place of IL-12 suggest that CAR-T cells engineered to secrete IL-18 enhances CAR-T cell survival and antitumor activity both *in vitro* and *in vivo* by producing IFN-γ and several other cytokines, stimulating expansion of human CD4^+^ cells as well as activating the endogenous immune system in immunocompetent mice (122, 123).

IL-15 is known to stimulate the generation of stem cell memory T cells (Tscm) with potential to sustain durable T cell responses (124). Unlike IL-2, IL-15 does not bind to the IL-2Rα chain, and thus does not stimulate Tregs and may have a more selective effect. When compared with IL2, IL15 tend to enhance CAR-T cell antitumor activity by preserving their Tscm phenotype (125). A comparison of CAR-T cell expansion in the presence of IL-2, IL-15, or a combination of IL-15/IL-7, revealed that IL-15 best enhances CAR-T persistence and function in a mouse model of multiple myeloma (126). Preclinical observations strongly support the antitumor activity of IL-15 mediated by CD8^+^ T cells (127), and IL‐15 co-expression in CD19-CAR-T not only revealed a strong killing effect against leukemia cells, but most of the persistent T cells were phenotypically consistent with Tscm that drive long‐term persistence (128).

To improve IL-15 half-life and effectiveness *in vivo*, IL-15 was associated with IL-15-receptor-α to form a pre-bound IL-15/IL-15Rα dimer, which showed stronger antitumor activity than IL-15 monomer (129). Recently, subcutaneous injection of recombinant human IL15 was tested in patients with advanced solid tumors, although the treatment produced substantial increases in circulating NK and CD8^+^ T cells, nonetheless, no objective responses were observed (130). However, when IL-15/IL-15Rα sushi-domain was co-expressed on CD5-specific CAR-T cells, and tested in a patient with relapsed T-lymphoblastic lymphoma with CNS infiltration, a rapid ablation of the CNS lymphoblasts to undetectable levels within 4 weeks and disease remission was observed (131). To address IL-15-induced immune checkpoint activation, IL-15 can also be combined with anti-PD-(L)1 and anti-CTLA-4 antibodies (132).

IL-21 is a newly discovered member of the common γ-chain family of cytokines. Like IL-12 and IL-15, and in contrast to IL-2, IL-21 does not stimulate Tregs, instead, it inhibits Treg expansion through suppression of Foxp3, thus favoring the enrichment of antigen-specific CD8^+^ T cells (133). IL-21 facilitates the maturation and enhances the cytotoxicity of CD8^+^ T cells and NK cells, and promotes the differentiation of memory CD8**^+^** T cells (134, 135). IL-21 synergizes when combined with IL-12 to further inhibit Tregs (136), and synergizes when combined with IL-15 to expand CD28-expressing antigen-specific CD8^+^ T cells (137, 138). Utilizing these characteristics, IL-21 performed much better than IL-2 or IL-15 during *in vitro* generation of antigen-specific CD8^+^ CTL and in an *in vivo* murine model of cancer immunotherapy (139, 140). In murine tumor models, intratumoral injection of IL-21 strongly inhibited tumor growth and increased the frequency of tumor-infiltrating CD8^+^ T cells and mice survival (141). In a phase 1/2 trial, 4 out of 4 leukemia patients who received WT1-specific CTL generated in the presence of IL-21 demonstrated both relapse-free survival without GvHD and did not need further anti-leukemic treatment (142).

To reduce the toxicity and increase the half-life of IL-21, IL-21 has been conjugated to tumor-targeting antibodies such as anti-EGFR antibody (143), selectively expanding functional CTLs while restricting exhausted T cells in the TME. IL-21 upregulates perforin and granzyme expression in memory and effector CD8^+^ T cells (144), thus augments the antitumor activity of CD8^+^ T cells (145), consistent with the requirement of IL-21 for the long-term maintenance and function of CD8^+^ T cells (146). IL-21 fused to anti-PD-1 antibody stimulated generation of Tscm with enhanced cell proliferation and tumor-specific CD8^+^ T cells, outperforming anti-PD-1 antibody and IL-21 infused as separate treatments (147). These results demonstrated that IL-21 can be used alone or in combination with other cytokines to produce tumor-specific T cells with a memory phenotype, with enhanced persistence, proliferative capacity, and antitumor efficacy for adoptive cancer immunotherapies.

Endogenous immune cells can act as a “sink” for administered cytokines (148), thus the use of a lymphodepleting conditioning regimen prior to ACT helps to spare the limited cytokines for the transferred T cells. Moreover, conditioning can also eliminate immunosuppressive Tregs and MDSCs (149), further supporting the engraftment and expansion of engineered T cells and improving therapy persistence and efficacy (150, 151).

Finally, purposeful selection of T cell sub-populations is another way to enhance persistence and functionality of the adoptively transferred T cells. Less differentiated T cells such as Tscm and central memory (Tcm) cells are more effective than effector T cells when transferred into tumor-bearing mice (152), thus CAR modification of naïve T cells can generate antigen-specific Tscm and Tcm cells with long *in vivo* persistence which mediated robust, long-lasting antitumor responses (153, 154). To preserve this early differentiated T cell population, tumor-specific CTLs can be stimulated by a combination of IL-21 and anti-CD3/anti-CD28 antibody-conjugated microbeads or nanomatrices (155, 156). Addition of IL-21 alone or in combination with other cytokines (such as IL-7 and IL-15) into the expansion culture, can further support the enrichment and expansion of Tscm cells with superior antitumor activity (157), consistent with recent clinical data that WT1-TCR engineered T cells generated in the presence of IL-21 showed long-lasting persistence with superior anti-leukemia activity in humans (13, 142).

### Enhancing the Homing and Penetration of Engineered T Cells Into Solid Tumors

To achieve tumor eradication, cancer-specific CTLs need to migrate and penetrate into solid tumors (158), driven by interactions between tumor-secreted chemokines and chemokine receptors expressed on CTLs (159–163). This process is rate-limited when CTLs express chemokine receptors at low density or that do not match the specific chemokines secreted by the tumors (164, 165). This creates an opportunity to engineer tumor-specific T cells’ chemokine receptors to match known chemokines or cytokines abundant in the TME, with encouraging results for enhanced CTL homing and antitumor efficacy.

The chemokines CCL2, CCL7, and CCL8 are expressed in many cancer types as well as cancer-associated fibroblasts (CAFs), tumor-associated macrophages (TAMs), MDSCs, and mesenchymal stem cells found in the TME, which support the tumor growth and metastasis (164). All three of these chemokines are ligands for the CCR2 receptor, thus when CCR2b was transduced together with a CAR specific for GD2 into human T cells, these modified T cells showed enhanced trafficking with >10-fold improved homing to CCL2-secreting neuroblastoma, and significantly enhanced activity against neuroblastoma xenografts *in vivo* (166). The same approach, resulted in 12.5-fold increase in infiltration of CAR-T specific for mesothelin into established mouse tumors, and significantly enhanced antitumor activity and tumor eradication (167). When CCR2 was transduced together with a TCR specific for WT1 into CD3^+^ human T cells, double gene-modified CD3^+^ T cells demonstrated CCL2-tropic tumor trafficking and potentiated antitumor activity against WT1-expressing LK79 lung cancer cells both *in vitro* and *in vivo* (168). Similarly, transduction of CCR2 into TCR-T cells specific for the SV40 large T antigen, enhanced recruitment into CCL2-expressing metastatic prostate adenocarcinoma, and improved *in vivo* antitumor effect (169).

Multiple chemokine ligands for the CXCR2 receptor are expressed in many tumors (170), and also promote tumor initiation, proliferation, migration, metastasis, and immune invasion. Thus, CXCR2 has been explored intensively for cancer immunotherapy. Human hepatocellular carcinoma (HCC) tumor tissues and cell lines express several chemokine ligands for CXCR2, however, both human peripheral T cells and TILs of HCC lack expression of CXCR2. In a recent study (171), Liu et al. transduced human T cells with a GPC3-CAR together with CXCR2; compared with CAR-T cells without CXCR2, these cells exhibited identical cytotoxicity but significantly increased migration *in vitro*, as well as accelerated *in vivo* trafficking and tumor-specific accumulation in a xenograft tumor model. Similarly, CXCR2 enhanced trafficking and *in vivo* antitumor efficacy of CAR-T cells specific for integrin αvβ6 in advanced pancreatic and ovarian tumor xenograft models (172). In the TCR-T field, when CXCR2 was transduced into pmel-1 TCR transgenic T cells (173), or MAGE-A3-specific TCR-engineered T cells (174), the CXCR2-TCR-T cells showed increased *in vivo* homing, enhanced tumor infiltration, and preferential accumulation in tumor sites in mice, with enhanced survival and tumor regression compared with mice receiving control TCR-T cells. These results indicate that introduction of the CXCR2 gene into tumor-specific T cells can enhance their homing and localization to tumors and improve antitumor immune responses. CXCR2 has also been used to enhance the migration and homing of NK cells to CXCL5-expressing renal cell carcinomas (175). Recently, Jin et al. used radiation therapy to induce tumor secretion of IL-8 (CXCL8), and found that CD70-CAR-engineered T cells expressing either of the IL-8 receptors CXCR1 or CXCR2, showed enhanced migration and persistence, leading to complete tumor regression and immunologic memory in models of aggressive tumors, including glioblastoma, ovarian, and pancreatic cancers (176). Like radiation therapy, chemotherapy may also induce chemokine secretion from tumor cells, resulting in increased homing and infiltration of adoptively transferred T cells (177). These studies indicate that genetic engineering of tumor-specific T cells with chemokine receptors can be combined with conventional radiation and chemotherapy to enhance antitumor efficacy. CXCR1 has also been used to enhance migration and tumor infiltration of NK cells modified with a CAR specific for NKG2D (178).

Other chemokine receptors used in this way include CCR4 and CXCR4. Similarly, coexpression of CCR4 enhanced migration of CD30-specific CAR-T cells in response to CCL17 secreted by Hodgkin’s lymphoma in murine xenografts (179). CXCR4 has also been explored as a means of recruiting T cells into the bone marrow, whose microenvironment is suggested to improve memory T cell formation and self-renewal. Khan (180) et al. overexpressed CXCR4 in CD8^+^ T cells, observing enhanced migration toward CXCL12-expressing cells in the bone marrow, with enhanced memory differentiation, expansion, persistence, and antitumor function of adoptively transferred T cells. CXCR4 also enhanced migration of NK cells to bone marrow as a means of targeting bone-marrow-resident tumor cells such as leukemia (181). CXCR4-modified CAR-NK cells also significantly improved survival and tumor regression of mice bearing glioblastoma (182).

Aside from engineering T cells with chemokine receptors, chemokines can be directly introduced into tumors to enhance T cell recruitment. For example, intratumoral injection of CXCL2 plasmid DNA combined with inactivated Sendai virus envelope, suppressed the growth of murine breast cancers and inhibited lung metastasis through recruitment of CTLs and neutrophils, further enhanced with anti-PD-1 antibodies to inhibit T cell exhaustion (183). Chemokines can also be introduced into tumors through chemokine-armed oncolytic viruses, which simultaneously, replicate within and directly kill tumor cells to amplify antitumor efficacy (184). T cells can also be engineered to express both chemokines and cytokines to improve antitumor efficacy. For example, transduction of CAR-T cells to express IL-7 and the chemokine CCL-19, not only enhanced T cell survival, infiltration and accumulation in the tumor, but also achieved complete regression of pre-established solid tumors and prolonged mouse survival (185). When the chemokine CCL21 and IL7 was transduced into CAR-T cells, it significantly improved survival and infiltration of both CAR-T and dendritic cells in the tumor, leading to complete tumor remission (186).

### Overcoming the Immunosuppressive Tumor Micro-Environment

Infiltration of genetically engineered T cells into the tumor is only the first step in fighting cancers. Tumor cells inhabit a heterogeneous microenvironment of infiltrating and resident host cells, secreted factors and extracellular matrix (187). Infiltrated cells include immune cells, such as T cells (TILs and Tregs), macrophages (M1 and M2), and MDSCs, and secreted factors include the immunosuppressive cytokines IL-10 and TGFβ. The TME also includes stromal cells such as CAFs and TAMs. These components can mutually interact to induce a supportive milieu for malignant cell growth, migration, and metastasis, that evades the immune system and tumor-specific CTLs (188, 189).

Most tumor stromal cells in the TME express the immunosuppressive checkpoint ligand PD-L1 (190–192), which can interact with PD-1 expressed on T cells, resulting in inhibition of antitumor function and exhaustion of adoptively transferred TILs (193), CAR-T (194) and TCR-T (195). This effect can be relieved *via* checkpoint blockade with anti-PD-1 (196–199) and anti-PD-L1 (200, 201) antibodies. CTLA-4 expressed on activated T cells have a similar effect, as CTLA-4 binds to CD80/86 on antigen-presenting cells with higher affinity in competition with the T cell costimulation molecule CD28, dampening antitumor immunity (202). Anti-CTLA4 antibodies both block the interaction between CTLA4 and CD80/86, and can also deplete Tregs (203), thus facilitate the costimulation and expansion of tumor-specific CTL with improved clinical benefits (204, 205).

Checkpoint inhibitors alone induce a response rate of approximately 20% of patients in one meta-analysis (206), and some responding patients will develop resistance (207). One important resistance mechanism is the upregulation of PD-L1 expression on tumor cells treated with immunotherapy, resulting in T cell exhaustion and relapse (207, 208). Immune checkpoint blockade is also associated with significant and in some cases life-threatening toxicity (209). An alternative approach to eliminating the immunosuppressive effect of PD-1 on tumor-specific CTLs uses CRISPR/Cas9 technology to remove PD-1 from CAR-T (210), and TCR-T cells (89). It is possible to go beyond PD-1-deletion by introducing a chimeric switch receptor (CSR) consisting of a PD-1 extracellular domain (PD1ex) and CD28 intracellular domain (CD28in). When this PD-1:CD28 CSR was transduced together with a CAR (211, 212) or TCR (213, 214) into T cells, the engineered CTLs still interact with PD-L1 on tumor cells, but delivers a costimulation signal *via* CD28 rather than an inhibitory signal. CAR-T cells generated using this strategy show increased cytokine production (211), enhanced killing ability, and increase in central memory T cells (212). Similarly,this PD-1:CD28 CSR enhanced TCR-T cells to increase cytokine production and cell proliferation *in vitro* and *in vivo* (213), and prevented PD-L1 upregulation and Th2 polarization in the TME (214). CSR TCR-T cells also synergized with anti-PD-L1 antibody to secrete more IFNγ compared with control TCR-T (214). Recently, this strategy has begun to be used in the clinic. In a CD-19 CAR-T study of relapsed/refractory diffuse large B-cell lymphoma (215), 6 patients who progressed following CD19-CAR-T therapy, were given CAR-T cells engineered with CSR PD1ex-CD28in, of which 3/6 patients achieved complete remission, and 1/6 achieved partial response. In another study, in relapsed/refractory PD-L1^+^ B-cell lymphoma (216), CSR-engineered CAR-T cells targeting CD19 showed superior T-cell proliferation, cytokine production, and cancer cell killing *in vitro* and *in vivo*. Among 17 treated adult patients, 10 patients had objective response (58.8%), including 7 with complete remission (41.2%). In both trials no severe neurologic toxicity or cytokine release syndrome was observed. Endogenous PD-1 was not depleted in these trials, thus we anticipate additional opportunity to enhance antitumor activity by combining CSR-engineered T cells with PD-1 knockout. The same CD28 CSR approach has also been applied to the immune checkpoint molecules TIGIT (T cell immunoreceptor with Ig and ITIM domains) and CTLA-4. Co-transduction of a TIGIT : CD28 CSR together with a tumor-specific TCR or CAR into human T-cells, drove enhanced cytokine production and superior anti-tumor function in a xenograft model of established human melanoma tumors (217). Transduction of a CTLA-4:CD28 CSR into tumor-specific T cells, resulted in elevated IFN-γ and IL-2 production and enhanced antitumor effect without systemic autoimmunity (218). A recent study engineered a CTLA4:CD28-CD3z CSR with the intracellular domains of both CD28 and CD3z, demonstrating increased cytokine production and cytotoxicity *in vitro* and in xenograft models (219). These engineered CAR-T cells were found to accumulate in tumors and to target MDSCs without severe GvHD or CRS (219).

Among the multiple immunosuppressive factors secreted within the TME, TGF-β plays a central role driving tumor signaling, remodeling, and metabolism (220). TGF-β is produced by many cell types including tumor cells, stromal cells and Tregs (221), and stimulates autocrine and paracrine signaling to promote angiogenesis (222), suppress CD8^+^ and T_h_1 anti-tumor responses (223), and induce epithelial-to−mesenchymal transition of neoplastic cells and thus facilitate tumor invasion (224). Recent clinical data associated patient non-responders to checkpoint blockade with TGF-β signaling (225). Therefore, blocking TGF-β signaling in the TME could potentiate antitumor responses. Indeed, one possible mechanism of anti-CTLA4 antibody therapy is the depletion of immunosuppressive TGF-β-producing Treg cells (203), thus facilitating the costimulation and expansion of tumor-specific CTL with improved clinical benefit (204, 205). To further improve on the efficacy of checkpoint inhibitor antibodies, bifunctional antibody-ligand traps comprising an antibody targeting CTLA-4 or PD-L1 fused to a TGFβ receptor II ectodomain (TGFβRIIecd) have been generated (226), in which TGFβRIIecd sequesters TGF-β secreted in the TME, while the checkpoint inhibitor antibody depletes Tregs and facilitates the CTL costimulation. This dual strategy may be more effective against cancers that are resistant to immune checkpoint inhibitors alone.

As an alternative to TGF-β sequestration, tumor-reactive T cells can be transduced with a dominant-negative TGFβ receptor-II (dnTGFβRII), generating TGF-β-resistant antitumor T cells (227). TCR-T cells expressing dnTGFβRII demonstrated complete tumor regression and prolonged survival in a mouse model of advanced and invasive prostate carcinoma. In a recent clinical study, patients with relapsed Hodgkin lymphoma were treated with EBV-specific T cells engineered to express dnTGFβRII, and 4/8 patients showed an objective clinical response (228), demonstrating that TGFβ-resistant tumor-specific T cells can persist safely in patients and potentially enhance the efficacy of T cell immunotherapy. To take advantage of the high concentration of TGF-β in the TME, a recent study fused the TGF-β receptor II (TGFβRII) extracellular domain to the intracellular domain of 4-1BB to convert the immunosuppressive effect of TGF-β into an immunostimulatory signal (229). The same cells were also transduced with a CAR-CD3ζ targeting the prostate-specific antigen and an inverted cytokine receptor consisting of the IL-4R extracellular domain fused to the IL-7R intracellular domain. Coexpression of these 3 transgenic receptors generated an additive effect with improved expansion, persistence, tumor lysis, and selective antitumor activity *in vivo*. Transduction of the TGFβRII-41BB CSR together with a NYESO1-specific TCR promoted abundant effector cytokine production in T cells, resulting in markedly enhanced tumor clearance in an *in vivo* solid tumor model (230). Like TGF-β, the Fas ligand-mediated T cell death signal that is highly expressed in the TME can also be converted into pro-survival signal *via* CSR, by fusing the Fas extracellular domain with the 4-1BB intracellular domain (231), resulting in engineered T cells with increased pro-survival signaling, proliferation, antitumor function, and enhanced *in vivo* efficacy against leukemia and pancreatic cancer mouse models. These studies clearly demonstrate the potential for using CSRs to convert the TME’s immunosuppressive signals into immunostimulatory signals in engineered antitumor T cells.

### Enhancing Tumor-Specific Killing by Targeting Neoantigens

Over the past decade, many tumor-associated antigens (TAAs) have been discovered and investigated as targets for cancer immunotherapy. These include the cancer testis antigens (e.g., New York esophageal squamous cell carcinoma 1 (NY-ESO)-1) (25), melanoma-associated antigen (MAGE-A3 (174), MAGE-A4) (232), differentiation antigens (e.g., melanoma antigen recognized by T-cells 1 (MART-1) (233), tyrosinase/gp100 (234), overexpressed oncogenes (e.g., Wilms’ Tumor antigen 1 (WT1) (13, 235, 236), surviving (237), tumor suppressor genes (e.g., TP53) (238); and TAAs that are organ-specific (e.g., prostate-specific antigens) (239) or cell-type-specific antigens that are transiently expressed during differentiation (e.g., terminal deoxynucleotidyl transferase) (240), or normally expressed only during embryonic development (e.g., carcinoembryonic antigen) (241). While many of these have advanced to the clinic, the residual expression of many TAAs in normal tissues often leads to toxicity from TAA-targeted therapy (234, 242–244). There is therefore a need to target truly tumor-specific antigens. This is the basis for targeting antigens from oncogenic viruses, such as HPV (245), EBV (246) and HBV (247), which can potentially eradicate virus-induced cancer cells (248, 249).

While most cancers are not viral in origin, they share a hallmark of genomic instability (250), which often leads to the occurrence of a large number of mutations. Mutant amino acid coding sequences can be expressed, processed, and presented on the surface of tumor cells as cancer-specific neoAgs, and subsequently recognized by T cells. CTLs targeting neoAgs are less likely to react against normal tissues or face immune tolerance. Indeed, evidence from treatment of cancers with checkpoint blockade suggests that tumors with higher mutational burden are likely to respond to immunotherapy (251, 252). In a study of 266 cancer patients, responders to checkpoint blockade therapy more often had tumors harboring TILs (so-called ‘hot’ tumors), while non-responders had tumors with few TILs (‘cold’ tumors) (253). It is thought that tumors harboring more mutations generate more neoAgs, which can be recognized by neoAg-specific TILs (254). These TILs are frequently suppressed in the TME by immune checkpoint molecules such as CTLA-4 and PD-1/PD-L1, but can be reactivated following checkpoint blockade and thus able to induce tumor regression. As a result, cancers with high mutational load, such as melanoma and lung cancer, are more susceptible to checkpoint blockade therapies. There is also evidence that checkpoint blockade not only increases the number but also enhances the antitumor activity of neoAg-specific TILs (255).

Combining these clinical data, we hypothesize that immunotherapy through checkpoint blockade could be further augmented with neoantigen vaccines. Such vaccines could stimulate and amplify neoAg-specific TILs, which are released and reactivated upon checkpoint blockade to destroy tumor cells. Indeed, a personalized RNA-based vaccine was recently used to treat stage III and IV melanoma patients (256). All 13 patients developed T cell responses against multiple neo-epitopes, and each patient developed T cells against at least three mutations. Vaccination reduced rate of metastases and sustained progression-free survival in 8 patients. Notably, 1 patient showed complete response when the vaccination was combined with PD-1 blockade therapy. In another study, 4/6 neoAg-vaccinated patients showed no tumor recurrence at 25 months after treatment, while 2/6 patients with recurrent disease subsequently showed complete tumor regression following treatment with anti-PD-1 therapy (257). A phase Ib trial combining a personalized neoAg vaccine with PD-1 blockade found durable, neoAg-specific CD4^+^ and CD8^+^ T cell responses in all 82 treated patients, and T cells migrated to metastatic tumors and mediated tumor cell killing (258). Many clinical studies suggest that neoAg-specific T cells are the main mediators of tumor destruction in patients who responded to checkpoint blockade therapy (252, 259) or adoptive T cell transfer (260, 261). Both CD8^+^ and CD4^+^ neoAg-specific T cells contribute to lasting tumor clearance (262–265), and work continues to develop strategies to promote maximal cytotoxic T cell responses (266).

## Summary and Future Perspective

**Figure 1:**
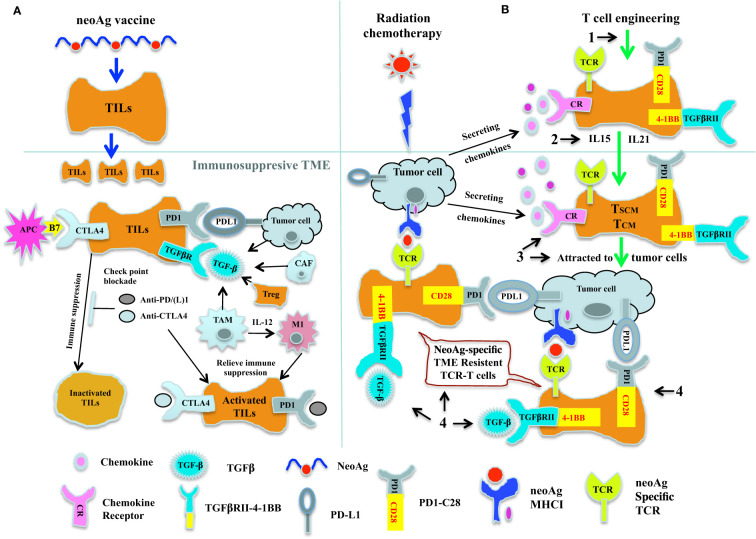
Cancer immunotherapy by combining neoAg vaccine and chekpoint blockade therapy together with adoptive transfer of neoAg-specific TCR-T. **(A)** Combination of neoAg vaccine and chekpoint blockade therapy. NeoAg vaccines can be used to stimulate and expand tumor reactive CTLs in the circulation system. When these expanded CTLs come into the TME, they will be inactivated by the check point molecules such as PDL1 and CTLA4, or by the immunosuppressive cytokine such as TGF-β (left). Using check point blockade therapies such as anti-PD(L)1 or anti-CTLA4 to block the check point interactions (middle) or using immunomodulatory cytokines such as IL-12 (right) to convert the tumor associated macrophage M2 into activated antitumor M1, these neoAg vaccine expanded CTLs can be reactivated and attack the tumor. **(B)** Genetically engineered TCR-T cells as a complement to neoAg vaccine and chekpoint blockade therapy. Radiation and chemotherapy can induce secretion of chemokines, which could potentially attract the tumor reactive CTLs to the tumor site. But more than offten, tumor reactive CTLs from patients are either too rare or with low avidity, thus could not control the tumor growth, as reflected by the fact that only a proportion of patients responded to neoAg vaccines or check point blockade therapies. Therefore, genetic engineering strategies could be used to complement the neoAg vaccines and check point blockade therapies. (1). By transducing patient’s T cells with neoAg-specific TCR, we could obtain truly tumor specific T cells. (2). Expanding these neoAg-specific TCR-T cells with IL-15 or IL-21, we could potentially acquire T cells with early differentiated phenotype of Tscm and Tcm. (3). By introducing chemokine receptor genes into these TCR modified T cells, these TCR-T cells could be attracted to the tumor site. (4). By expressing chimeric switch receptor (CSR) on these neoAg-specific TCR-T cells, the immunosuppressive effect of certain immune suppression factors such as PD-L1 or TGF-β within the TME could be potentially converted into immunostimulatory signals inside these TCR-T cells. Thus, with these innovative engineering strategies, we could not only obtain sufficient numbers of high avidity, early differentiated long lasting tumor reactive TCR-T cells, but these T cells could also be attracted and infiltrate into the solid tumor, and within the TME, these TCR-T cells would have the ability not only to resist the the immunosuppressive effect of the TME, but could also get stimulated and further expanded by neoAg vaccine and check point blockade therapies, and finally achieve the ultimate goal of destroying the tumor.

The power of the human immune system in fighting cancer has been demonstrated by the adoptive transfer of TILs (1, 2) and CAR-T therapy for hematological malignancies (5, 6). However, the promise of adoptive cell therapy for solid tumors has not yet been fully realized (14, 15). TCR-T therapy holds a number of advantages over alternative strategies. TCRS can recognize epitopes derived from both surface and intracellular proteins, enabling detection of a much broader range of targets compared to CAR-T, including TAAs, cancer germline antigens, viral oncoproteins, and neoAgs. Moreover, TCRS have naturally developed to sensitively detect low epitope concentrations, down to as little as a single molecule. Recent clinical research on TCR-T has produced meaningful responses in a variety of cancers (24–27), and in some cases durable and curative responses in solid tumor patients (29, 262–265). However, as many of these studies mainly targeted TAAs, we envisage targeting tumor-specific neoAgs to produce more profound antitumor immune responses. To achieve the goal of complete eradication of solid tumors, several aspects need to be considered:

1. Targeting tumor-specific antigens, combining neoAg vaccines, checkpoint blockade therapy, and adoptive transfer of genetically engineered neoAg-specific T cells (**Figure 1**). While targeting neoAgs can achieve complete tumor regressions in some settings, combination strategies are likely to expand their utility and increase response rates. We propose to extend the concept of combining neoAg vaccines and checkpoint blockade therapy, together with the adoptive transfer of neoAg-specific T cells as a generalizable therapeutic strategy. This starts with high-throughput screening of large patient cohorts – encompassing multiple tumor types – to identify and collect a library of patient-derived neoAgs. This will enable vaccination using combinations of unique and shared cancer-specific neoAgs, to be tested in conjunction with checkpoint blockade therapies. In a second step, we propose to isolate neoAg-specific CTLs from responding patients, especially the CTLs specific for neoAgs representing driver mutations, to construct a TCR library that can be used to generate cell therapies for patients where vaccination is not effective (267). Challenges faced in this step include the limited availablity of patient derived materials and the low frequencies of neoAg-specific T cells from patients. To overcome these limitations, Stronen et al. developed a strategy for the induction and isolation of neoAg reactive T cells from healthy donor T cell repertoires (268), which contain higher frequency of neoAg reactive T cells that are not affected by the patient’s TME. Recently, this strategy has been optimized by Ali et al. into a standard protocol which will facilitate the isolation of neoAg specific T cells for cancer immunotherapy (269).
2. Eliminating TCR mis-pairing. Although neoAgs represent ideal targets for cancer immunotherapy, nonetheless, they are frequently ignored by patient’s TILs (268). Under such situation, adoptive transfer of high avidity neoAg-specific TCR-T cells will be a valuable supplementation to the neoAg vaccine and check point blockade therapies. However, mis-pairing of introduced TCR with endogenous TCRS could potentially cause auto-reactivity against patient’s MHC molecules, thus thoughtful innovations in TCR engineering technology could be incorporated. In this regard, genetic engineering of the TCR constant domains can be combined with design of a single-chain TCR, and the CRISPR/Cas9 genome editing can be used to orthotopically replace the endogenous TCR with tumor reactive TCR (83, 84). Through applying these recent innovative technologies in T cell engineering, TCR mis-pairing can be eliminated, while generating antitumor T cells with enhanced TCR expression and functions.
3. Maintaining long-lasting immunosurveillance against tumors and keeping patients in relapse-free survival. To achieve this goal, a fundamental requisite is persistence of genetically engineered T cells after ACT. Provision of cytokines can play important roles in supporting T cell survival and functions (99, 100, 137, 138), but is often associated with severe cytotoxicity if delivered systemically (113). Therefore, controlled and targeted delivery of cytokines through genetic engineering of tumor-specific T cells (116–119), can not only support T cell survival and generate long-term memory T cells (124, 138), but can also modify the TME to create an inflammatory environment (109, 111), and maintain a “hot” tumor milieu that self-sustains the antitumor immune responses (120).
4. Facilitating migration and penetration of genetically engineered T cells into the solid tumor (158). Genetic engineering of tumor-specific T cells with chemokine receptors that match chemokines secreted by the TME can be adopted to recruit T cells to the tumor sites (168, 174). For enhanced antitumor effect, chemokines and cytokines can be combined (185, 186), and introduced through oncolytic viruses or vaccine adjuvants (159, 184). Radiation and chemotherapy can further augment ACT by stimulating chemokine secretion from tumor cells, increasing homing and infiltration of adoptively transferred T cells into solid tumors with enhanced antitumor activity (176, 177).
5. Overcoming the immunosuppressive effects of the hostile TME and fully realizing the antitumor potential of engineered T cells. The TME represents a formidable hostile environment for antitumor T cells and favors tumor growth, metastasis, and immune evasion. The field has made advances in blocking the immunosuppressive factors of the TME (206), and developed innovative genetic engineering strategies to convert immunosuppressive ligands/factors into immunostimulatory signals (211–214, 229). These strategies can not only remodel the immunosuppressive network within the TME (230), and convert ‘cold’ tumors lacking TILs into ‘hot’ tumors with genetically engineered T cells, but can also enhance T cell co-stimulation and survival, and produce TME-resistant antitumor T cells (214, 231, 270). These TME-resistant T cells can be further expanded by neoAg vaccines, and their antitumor activity can be further enhanced by the checkpoint blockade therapies, and potentially lead to complete tumor eradication.

With the fast development and innovation of genetic engineering technologies, incorporating the aspects summarized above into the TCR-T therapeutics development, TCR-engineered T cells can be made truly tumor-specific and have the ability to migrate and penetrate into solid tumors, and become TME-resistant. TCR-T has potential to become a powerful tool for fighting cancers, especially solid tumors where other approaches have been less effective. By combining neoAg vaccines, checkpoint blockade therapy, and the adoptive transfer of neoAg-specific TCR-engineered T cells, we believe such a combination approach could lead to significant improvement in cancer immunotherapies, and this approach is scalable across different tumor types, and may provide a general strategy for the eradication of multiple cancers.

## Author Contributions

All authors listed have made a substantial, direct and intellectual contribution to the work, and approved it for publication.

## Funding

This work was supported by the National Natural Science Foundation of China (NSFC-81972883); Social Development & Scientific Technology Key Project of Shaanxi Province (2016SF-079); and Scientific Technology Program & Innovation Fund of Xi’An City Special Project (CXY1531WL12).

## Conflict of Interest

SAX is an inventor on patents describing a Sc-TCR technology.

The remaining authors declare that the research was conducted in the absence of any commercial or financial relationships that could be construed as a potential conflict of interest.

## Publisher’s Note

All claims expressed in this article are solely those of the authors and do not necessarily represent those of their affiliated organizations, or those of the publisher, the editors and the reviewers. Any product that may be evaluated in this article, or claim that may be made by its manufacturer, is not guaranteed or endorsed by the publisher.

## Abbreviations

CAR, Chimeric antigen receptor; CDR3, Complementarity-determining region 3; Cα: Constant alpha; Cβ: Constant beta; CRISPR, Clustered regularly interspaced short palindromic repeat; CRS: Cytokine release syndrome; CSR, Chimeric switch receptor; CTL, Cytotoxic T-Lymphocyte; CTLA4: Cytotoxic T-Lymphocyte Associated Antigen 4; EBV, Epstein-Barr Virus; GvHD, Graft-vs-host disease; HLA, Human leukocyte antigen; IFNγ, Interferon gamma; IL2, Interleukin 2; LCK, Lymphocyte-specific protein tyrosine kinase; mCα, Murine Constant alpha; MDSC, Myeloid-derived suppressor cells; MHC, Major histocompatibility complex; PBMC, Peripheral blood mononuclear cell; PLK, Polylinker; Sc, Single chain; Sc-TCR, Single-chain T-cell receptor; siRNA, Small interfering RNA; sgRNA, Single guide RNA; TAA, Tumor associated antigen; TALENs, Transcription activator-like effector nucleases; Tcm, Central memory T cells; TCR, T-cell receptor; TGF-β; Transforming growth factor-β; TNFα, Tumor necrosis factor-alpha; Treg, Regulatory T cells; TRAC, T cell receptor constant-alpha; TRBC, T cell receptor constant-beta; Tscm, Stem cell memory T cells; WT1, Wilms’ tumor 1.
